# Loss of audiovisual facilitation with age occurs for vergence eye movements but not for saccades

**DOI:** 10.1038/s41598-022-08072-9

**Published:** 2022-03-15

**Authors:** Martin Chavant, Zoï Kapoula

**Affiliations:** IRIS Laboratory, Neurophysiology of Binocular Motor Control and Vision, CNRS UAR 2022, University of Paris, 45 Rue des Saints Pères, 75006 Paris, France

**Keywords:** Cognitive ageing, Cognitive neuroscience, Motor control, Neural ageing, Oculomotor system, Sensory processing

## Abstract

Though saccade and vergence eye movements are fundamental for everyday life, the way these movements change as we age has not been sufficiently studied. The present study examines the effect of age on vergence and saccade eye movement characteristics (latency, peak and average velocity, amplitude) and on audiovisual facilitation. We compare the results for horizontal saccades and vergence movements toward visual and audiovisual targets in a young group of 22 participants (mean age 25 ± 2.5) and an elderly group of 45 participants (mean age 65 + 6.9). The results show that, with increased age, latency of all eye movements increases, average velocity decreases, amplitude of vergence decreases, and audiovisual facilitation collapses for vergence eye movements in depth but is preserved for saccades. There is no effect on peak velocity, suggesting that, although the sensory and attentional mechanisms controlling the motor system does age, the motor system itself does not age. The loss of audiovisual facilitation along the depth axis can be attributed to a physiologic decrease in the capacity for sound localization in depth with age, while left/right sound localization coupled with saccades is preserved. The results bring new insight for the effects of aging on multisensory control and attention.

## Introduction

Eye movements provide an ideal tool to interpret and understand the brain’s mechanisms involved in perception and action. The neuronal networks underlying these movements are well-known in humans, as it is one of the best-understood sensorimotor systems. Much of our understanding of sensorimotor control in neural processes arises from our knowledge of the oculomotor system as a microcosm of human behavior. Therefore, studying how age affects eye movements can be linked to alterations in the function of particular brain areas and pathways. Physiological brain aging is characterized by a loss of sensory processing, motor performance, and cognitive function caused by loss of synaptic contact^1^. Studies with magnetic resonance imaging (MRI) and diffusion tensor imaging (DTI) confirm this loss. The frontal and parietal cortex, which participates in saccades and vergence movement initiation, shows progressive degeneration with age^2,3^.

The saccades and vergence are types of eye movements allowing the exploration of the environment in three dimensions. Vergence eye movements enable the body to adjust the angle of the optic axes as a function of depth (i.e., to increase the convergence angle for near objects and to decrease it for distant objects); this is a prerequisite for obtaining bi-foveal single vision of objects located in different depths. Studies examining the effects of aging on both saccades and vergence are scarce.

For horizontal saccades, several studies show an increase in latency and a decrease in velocity and accuracy with age when comparing young and elderly adults^4–7^. In terms of vergence, Rambold^8^ reported an increase in latency and decrease of peak velocity and of acceleration with age. Yang et al.^9,10^ also reported an increase in latency but did not demonstrate an aging effect for peak velocity of vergence. However, they did show an increase in duration which was attributed to slowing of the velocity during the deceleration phase of the vergence movement. Yang et al. used a relatively small population (10 vs 13) to conclude that aging affects mostly visual sensory processing, e.g., the binocular disparity necessary to drive the vergence movement to the end rather than the motor component itself. Thus, there are contradictions between different studies on vergence, perhaps related to the small populations studied.

Our first aim is to bridge this gap by studying aging of all spatiotemporal parameters of both saccades and vergence eye movements in a larger healthy population ranging from 50 to 84 years old.

Our second goal is to provide new data on possible audiovisual facilitation, as, in real life, most stimuli of vergence and saccades are audiovisual. In general, multimodal stimuli create a multisensory facilitation of movements, leading to better, faster responses. These different modalities need to be merged at the cortical level to elicit a motor programming signal. Multisensory facilitation is known to improve manual reaction time^11^. It has also been studied in saccade reaction time: an auditory cue that is spatially and temporally aligned with a visual target decreases saccade preparation time^12–14^. The time window inside which this multisensory facilitation occurs ranges from 46 to 600 ms^15,16^. This facilitation is either due to an interaction at a multimodal stage of saccade programming and/or to a warning aspect of the auditory accessory unspecific to location. The warning aspect seems more plausible as the facilitation effect is more important when the auditory accessory is presented slightly before the visual target^11^. The superior colliculus (SC), a neuronal structure lying in the midbrain, is presumed to play an essential role in this multisensory facilitation. It is involved both in the initiation and the execution of saccades^17^ and also in the auditory pathway^18^. The SC cells, especially saccades relative burst neurons, exhibit profound changes in their activity when auditory, visual, or somatosensory stimuli are combined^13,19^.

Concerning the literature on the audiovisual facilitation for eye movements, until now most studies have focused on saccades, with very few examining vergence. Two small case studies in young persons showed for the first time different audiovisual effects on eye movement between saccades and vergence. Namely, for saccades, the addition of a sound preceding the visual target reduces saccade latency; it simultaneously accelerates velocity in vergence. For movements combining saccades and vergence, mixed-effects are shown, namely on the latency of either component saccades or vergence^20,21^. Moreover, the psychophysical study by Kapoula and Chavant shows that hearing amplification reduces the latency of eye movements to visual targets. Such preliminary but pioneering studies reveal the interest of a novel way of investigating eye movements, considering multisensory aspects.

The third goal of this study is to examinate the aging effect of audiovisual facilitation on both saccades and vergence eye movements. There is no study exploring oculomotor movements towards audiovisual targets depending on age. Given the above-cited, albeit limited, literature, one should expect aging effects on multisensory processing that could be specific to the type of eye movements (saccades vs vergence).

We will therefore assess the latency, peak velocity, average velocity, and gain amplitude of saccades and vergence eye movements towards visual targets (LEDs alone) and audiovisual targets (LEDs and buzzer). Studying the responses to multimodal targets across age for saccade and vergence could help understand the neural processing that is the basis of this multisensory integration and response.

## Results

### Latency

**Figure 1:**
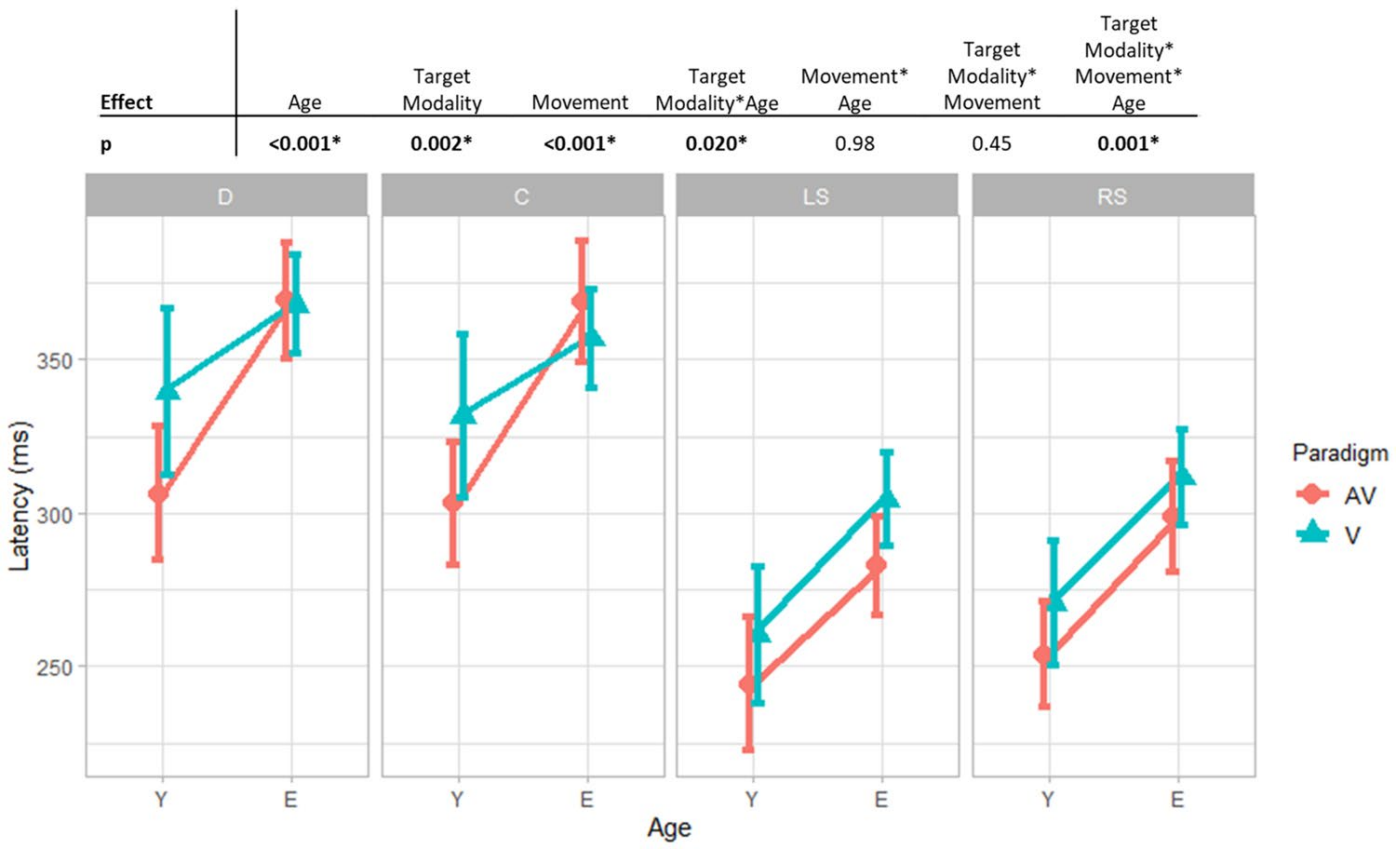
Summary of the main and interaction effects of the 3-way ANOVA with repeated measures for latency. Latency means and 0.95 confident intervals in function of movement (D for divergence, C for convergence, LS for left saccade, RS for right saccade), age group (Y for young group, E for elderly group) and modality target (AV for audiovisual target, V for visual target).

Group means and standard deviations are given in Table 1. Graphics and effects of the 3-way ANOVA are given in Fig. 1.

**Table 1:** Latency means and standard errors.

AV				V				Mean AV and V			
D	C	LS	RS	D	C	LS	RS	D	C	LS	RS
**Y, n = 22**											
306 (14)	303 (13)	244 (12)	254 (10)	339 (13)	332 (12)	260 (11)	271 (11)	323 (12)	317 (11)	252 (11)	262 (11)
277 (9)				300 (8)				289 (8)			
**E, n = 45**											
373 (9)	371 (9)	282 (8)	299 (9)	368 (9)	356 (9)	304 (8)	312 (8)	370 (8)	364 (8)	293 (7)	306 (7)
331 (6)				335 (6)				332 (7)			
**Y and E, n = 67**											
340 (8)	337 (8)	263 (7)	277 (7)	353 (8)	344 (8)	282 (7)	291 (7)	346 (7)	341 (7)	273(6)	284 (7)
304 (5)				318 (5)							

Means and sd in ms of the latencies, by group age, movements and targets modalities.

Y for the young group. E for the elderly group. A for auditory targets, AV for audiovisual targets. D for divergence, C for convergence, LS for left saccades, RS for right saccades.

There are significant main effects of age (*p* < 0.001) and target modality visual versus audiovisual (*p* = 0.002). The latency is significantly longer in the elderly group as compared to the young group (mean 332 ms with a standard deviation of 7 ms vs mean 289 ms with a standard deviation of 8 ms) and longer toward the visual targets than toward the audiovisual targets (318 ± 5 ms vs 304 ± 5 ms). The results also show significant interactions between the target modality and age (*p* = 0.020) and between age, movement, and target modality (*p* = 0.006). As seen in Fig. 1, for convergence and divergence for the elderly group, there is no latency difference between movements toward visual and audiovisual targets. In sum, the results on latency show: (1) an audiovisual facilitation mediated by the buzzer signal for the audiovisual target, (2) a deterioration of latency with age, (3) movement-specific deterioration of audiovisual facilitation.

### Peak velocity and average velocity

**Figure 2:**
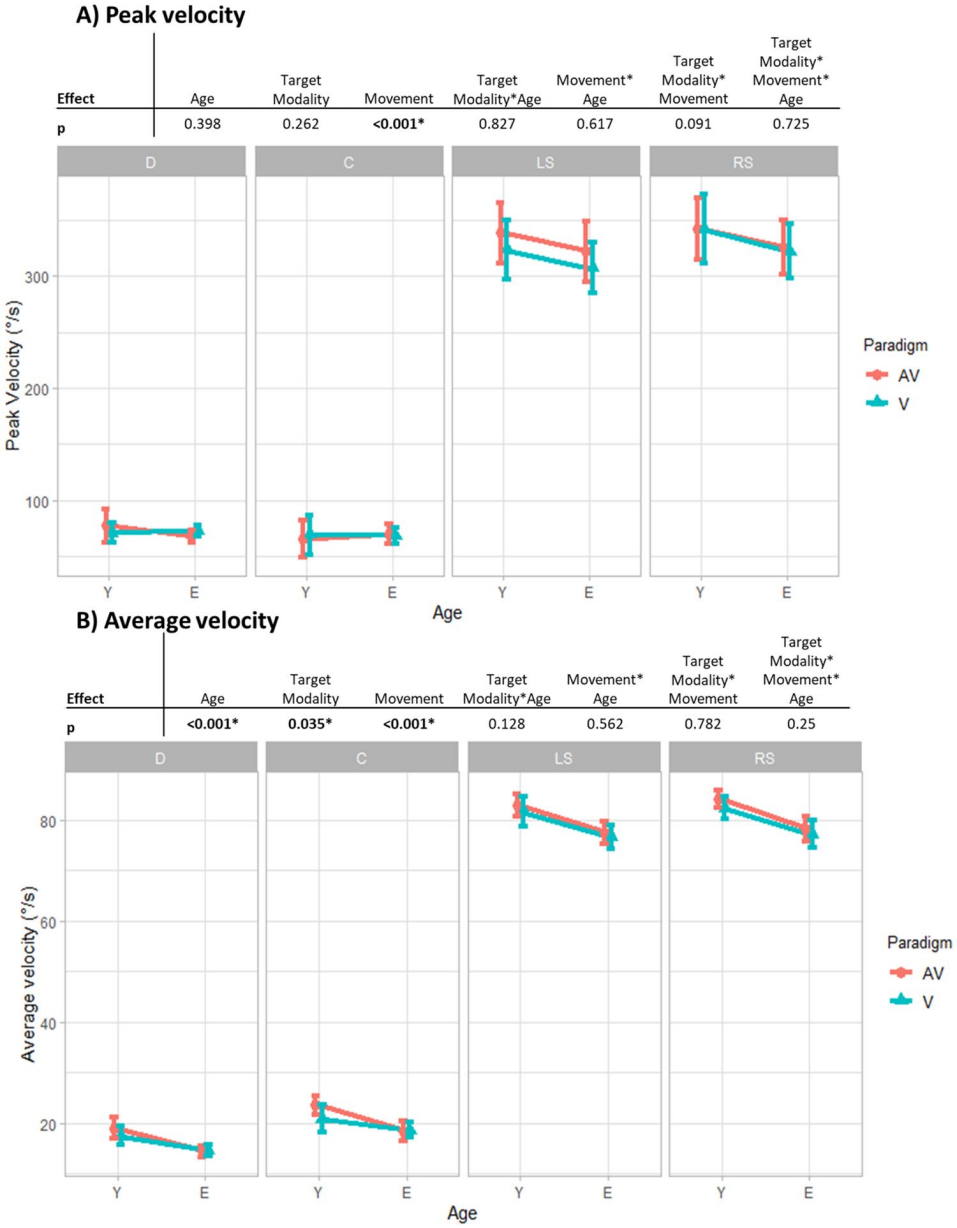
Summary of the main and interaction effects of the 3-way ANOVA with repeated measures for velocity (**A** for peak velocity and **B** for average velocity). Velocity means and 0.95 confidence intervals given as a function of movement (D for divergence, C for convergence, LS for left saccade, RS for right saccade), age group (Y for young group, E for elderly group) and modality target (AV for audiovisual target, V for visual target).

Group means and standard deviations are given in Table 2A for peak velocity and Table 2B for average velocity. Graphics and effects of the 3-way ANOVA are given in Fig. 2.

**Table 2:** Velocity means and standard errors.

AV				V				Mean AV and V			
D	C	LS	RS	D	C	LS	RS	D	C	LS	RS
**(A) Peak velocity**											
Y, n = 22											
77 (5)	65 (7)	339 (18)	343 (17)	71 (4)	69 (7)	324 (16)	342 (17)	74 (4)	67 (7)	331 (16)	343 (16)
206 (9)				201 (8)				204 (8)			
E, n = 45											
68 (4)	70 (5)	324 (13)	326 (12)	73 (3)	71 (5)	308 (11)	324 (12)	71 (3)	70 (5)	316 (11)	325 (11)
197 (6)				194 (6)				196 (5)			
Y and E, n = 67											
72 (3)	68 (4)	331 (11)	334 (10)	72 (2)	70 (4)	316 (10)	333 (11)	72 (2)	69 (4)	324 (10)	334 (10)
201 (5)				198 (5)							
**(B) Average velocity**											
Y, n = 22											
19.1 (1)	23.6 (1.3)	83.0 (1.5)	84.2 (1.6)	17.5 (0.9)	21.0 (1.2)	81.8 (1.7)	82.5 (1.8)	18.3 (0.8)	22.3 (1.1)	82.4 (1.5)	83.4 (1.6)
52.5 (1.0)				50.7 (1.1)				51.6 (1.0)			
E, n = 45											
14.4 (0.7)	18.4 (0.9)	77.5 (1.0)	78.2 (1.1)	14.7 (0.6)	18.8 (0.9)	76.6 (1.2)	77.2 (1.2)	14.5 (0.6)	18.6 (0.8)	77.0 (1.1)	77.7 (1.1)
47.1 (0.7)				46.8 (0.8)				47.0 (0.7)			
Y and E, n = 67											
16.8 (0.6)	21.0 (0.8)	80.2 (0.9)	81.2 (1.0)	16.1 (0.5)	19.9 (0.7)	79.2 (1.0)	79.9 (1.1)	16.4 (0.5)	20.4 (0.7)	79.7 (0.9)	80.5 (1.0)
49.8 (0.6)				48.8 (0.7)							

Means and sd in °/s of the peak velocity (A) and average velocity (B), by group age, movements and targets modalities.

Y for the young group. E for the elderly group. A for auditory targets, AV for audiovisual targets. D for divergence, C for convergence, LS for left saccades and RS for right saccades.

There are no significant differences in peak velocity based on age or the target modality, nor a significant interaction effect. This result is important as it demonstrates that there are no aging effects on peak velocity for neither saccades nor for vergence.

There are significant main effects of age (*p* < 0.001) and target modality (0.035). Average velocity is significantly lower for the elderly group than for the young group (47.0 ± 0.7°/s vs 51.6 ± 1.0°/s) and higher toward the audiovisual targets than toward the visual targets (49.8 ± 0.6°/s vs 48.8 ± 0.7°/s). No significant interaction effects were observed.

In summary, the initial peak velocity was not affected by age; however, the average velocity, which includes both the acceleration and deceleration phases of the eye movements, was slower in the elderly group.

### Gain amplitude

**Figure 3:**
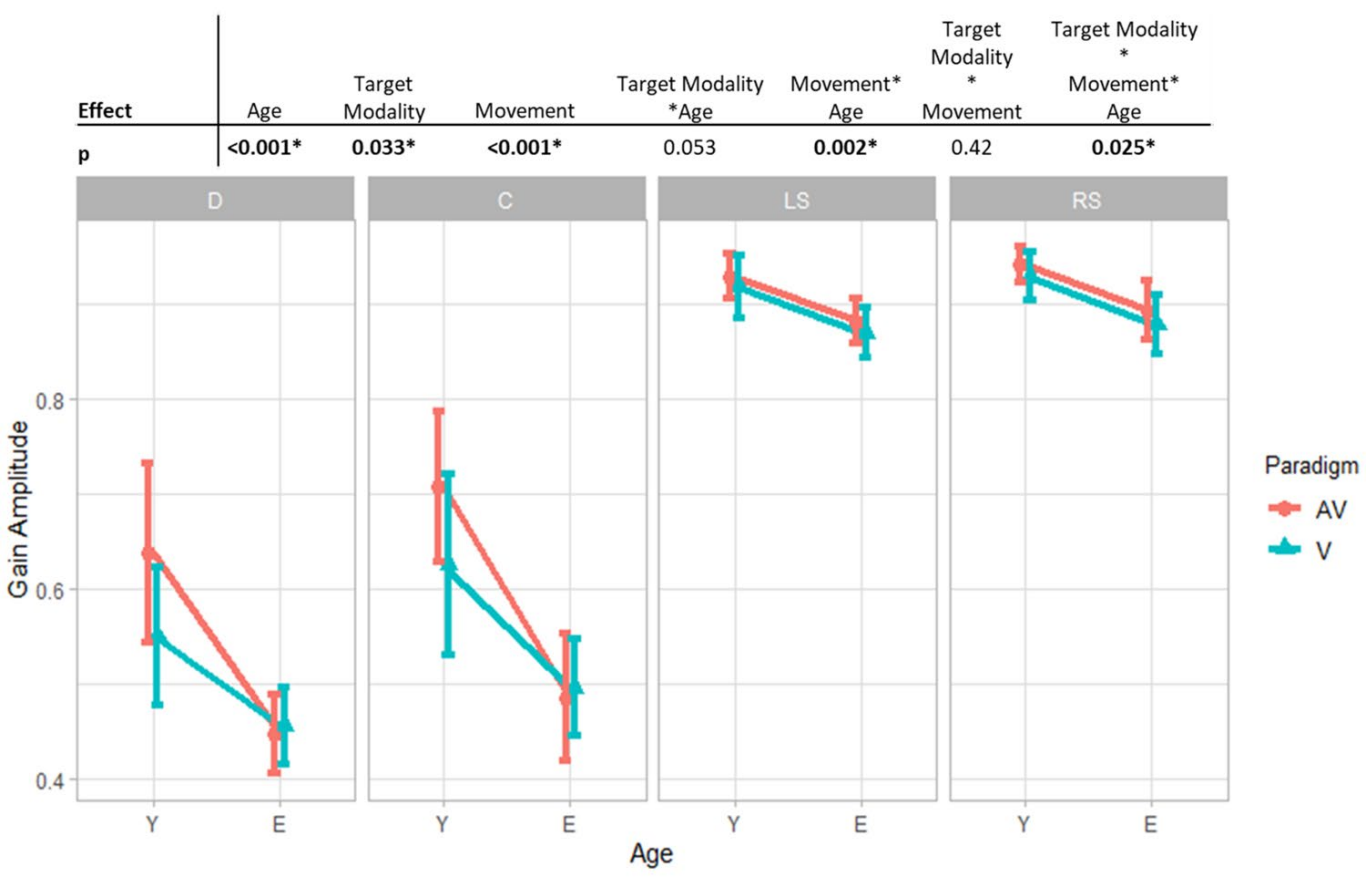
Summary of the main and interaction effects of the 3-ways ANOVA with repeated measures for gain amplitude. Gain amplitude means and 0.95 confidence intervals in function of movement (D for divergence, C for convergence, LS for left saccade, RS for right saccade), age group (Y for young group, E for elderly group) and modality target (AV for audiovisual target, V for visual target).

Group means and their standard deviations are given in Table 3. Graphics and effects of the 3-way ANOVA are given in Fig. 3.

**Table 3:** Gain amplitude means and standard errors.

AV				V				Mean AV and V			
D	C	LS	RS	D	C	LS	RS	D	C	LS	RS
**Y, n = 22**											
63.7 (3.7)	70.7 (4.17)	92.9 (1.6)	94.3 (2.0)	55.0 (3.3)	62.4 (4.2)	91.9 (1.9)	93.0 (2.0)	59.3 (3.0)	66.6 (3.9)	92.4 (1.6)	93.6 (1.9)
80.4 (2.1)				75.6 (2.1)				74.0 (1.3)			
**E, n = 45**											
44.4 (2.6)	48.6 (3.3)	88.0 (1.4)	89.2 (1.1)	45.4 (2.3)	49.3 (2.9)	86.8 (1.3)	87.8 (1.4)	44.9 (2.1)	49.0 (2.7)	87.4 (1.1)	88.5 (1.3)
67.6 (1.5)				67.3 (1.5)				71.4 (1.3)			
**Y and E, n = 67**											
54.1 (2.3)	59.6 (2.9)	90.5 (1.0)	91.7 (1.2)	50.2 (2.0)	55.9 (2.6)	89.3 (1.1)	90.4 (1.2)	52.1 (1.9)	57.8 (2.4)	89.9 (1.0)	91.1 (1.1)
78.0 (1.9)				67.4 (1.3)							

Means and sd in % of the gain amplitude, by group age, movements and targets modalities.

Y for the young group. E for the elderly group. A for auditory targets, AV for audiovisual targets. D for divergence, C for convergence, LS for left saccades, RS for right saccades.

Recall that the gain amplitude is estimated as the ratio of eye movement amplitude over the target amplitude requirement. Gain values are thus measured between 0 and 1, with 1 representing 100% accuracy. Gain amplitude was lower for the elderly group than young group (74 ± 1.3% for the young group, and 71.4 ± 1.3% for the elderly group; *p* < 0.001) and higher for the movement toward audiovisual targets than toward visual targets (78.0 ± 1.9% vs 67.4 ± 1.3%; *p* = 0.033).

Significant interactions were also found between movement and age (*p* = 0.002) and between age, movement and target modality (*p* = 0.025). Such significant interactions indicate that audiovisual targets improve gain amplitude only for vergence eye movements and only in the young group.

To summarize, age decreases the gain amplitude of eye movement, mostly for vergence. There is an audiovisual facilitation that increases gain amplitude for vergence movements only and only in the young group; such facilitation disappears with age.

## Discussion

This study shows that age has significant effects on eye movements. Most importantly, it shows that these effects differ according to different characteristics of the eye movement (latency, velocity, gain amplitude), type of eye movement (vergence vs saccades), and modality of the targets (visual vs audiovisual). Thus, the first overall conclusion is that aging selectively affects some aspects of eye movement control and is not a generalized phenomenon.

We will discuss the importance of the data for each parameter as presented in the results. Recall that latency is dependent upon cortical networks responsible for the preparation of eye movements, while velocity reveals mostly the quality of function of the subcortical movement generators located at the brainstem. Finally, the gain, or accuracy of the eye movement, reveals the quality of functioning of both cortical and subcortical circuits^22^.

### Aging effect depending on the movement

#### Preparation of the movement (latency)

As previously mentioned, eye movement latency is related to cognitive executive function^8–10^. The latency process comprises several steps: release of ocular fixation, shift of visual attention, computation of the eye movements metrics, and the decision to move the eyes. Many cortical areas, including frontal and parietal cortices, take part in this process.

The present study demonstrates an increase in latency during both saccades and vergence with age. The literature regarding the consequences of aging on latency is more robust for saccades than vergence. Many articles have demonstrated that latency of saccades increases with age^4,6,7,23,24^. In terms of vergence, the few existing studies also report significantly longer latency in elderly as compared to younger groups^8,9^. Our results are consistent with the literature on saccades and reinforce the few previous reports on vergence latency.

Our finding that latency increases with age in saccades and vergence can plausibly be attributed to the age-related progressive degeneration of the frontal and parietal cortex, where the initiation of saccades and vergence is programmed^2,3^.

#### Velocity of the movement

We found that average velocity decreases with age in both saccades and vergence, while peak velocity remained the same in both groups.

The literature on this topic is inconsistent for both saccades and vergence, given that most studies examine peak velocity alone. For saccades, Pitt and Rawles tested velocity for 85 participants aged 20–68 and found a decrease by about 0.25% per year^7^, while Munoz found no difference between the velocity of saccades with 20° eccentricity in young adults and older people^6^. For vergence, the two studies focusing on the effects of age on peak velocity were contradictory. According to Rambold^8^, the peak velocity of vergence for the elderly group (61 years ± 6) was significantly lower than for the young group (25.7 years ± 4). In contrast, the study of Yang and Kapoula^10^ did not find significant differences between peak velocity of vergence for their young group (25 ± 3) and their elderly group (70 ± 11). However, the latter study found a longer total duration of the vergence, suggesting a lower velocity during the decelerating, visually-driven part of the movement. Similar to Yang and Kapoula, the present study shows a difference in average velocity but not in peak velocity between the elderly group and the young group. This is in line with the idea that age does not affect the basic ocular motricity, but rather affects visual processing during the closed-loop component of vergence^10,25^. The findings of the current study are also consistent with previous studies demonstrating no neuronal loss due to normal aging in the area of the midbrain where the premotor neurons initiating vertical gaze belong, i.e., the medial longitudinal fasciculus (riMLF)^26^. The vergence tonic cells, which lie in the mesencephalic reticular formation of the midbrain, discharge based on the angle of vergence^27,28^. May et al. found that the profile, the number of spikes, and the firing rate of the vergence burst cells in the midbrain are correlated with instantaneous vergence velocity and vergence movement size^29^.

#### Gain amplitude of the movement

The results show a diminution of the gain amplitude with age. The existing literature is inconclusive with regards to the effect of age on the gain amplitude of saccades. Munoz et al.^6^ did not find a significant difference between the gain amplitude of saccades for young adults and elderly people. However, Irving et al.^30,31^ found that vertical and horizontal saccades were more accurate in the young adult population than the elderly. Concerning the vergence, Yang and Kapoula^10^ found no difference between the gain amplitude of vergence in young adults and older people. Unfortunately, in their study, the gain amplitude, including later visually-driven components, was estimated, while in the current study, the amplitude measured for vergence was stopped at 160 ms after the initial component. Our observations are consistent with the idea that age decreases the velocity of the visually driven component (or the time period 160 ms after initiation), which results in a smaller gain amplitude. In other words, the smaller gain amplitude measured during this period is a manifestation of the slowed visual feedback driving the execution of the movement.

Next, we will discuss the most intriguing results concerning the effect of multisensory stimulation.

### Audiovisual facilitation

Our finding that saccade latency is reduced in response to audiovisual targets as opposed to visual targets alone is consistent with previous literature and attributed to audiovisual facilitation^12–14,32^. Beyond the existing literature, our study shows for the first time that this audiovisual facilitation for saccade latency is preserved with age. However, there is one interesting exception: latency in vergence movements to audiovisual targets decreases with age. The fact that the audiovisual facilitation persists with age only on saccades and not for vergence demonstrates selective multisensory aging.

The superior colliculus is a major site of audiovisual facilitation; the multimodal cells in the deep layers of superior colliculus have a firing rate that is significantly higher for multimodal stimuli than for unimodal stimuli^19^. The latency effects observed here could be mediated by a differential effect of aging on these multimodal neurons of the superior colliculus, which respond differently based on the type of eye movement.

### Auditory perception of distance in depth

Another possible hypothesis concerning this loss of audiovisual facilitation for vergence with age could be linked to loss of hearing ability in the elderly population, leading to a suppression of audiovisual facilitation by the auditory signal.

Presbycusis is a notable decrease in hearing acuity with age, usually beginning in the fifth decade of life. It is characterized by an increase of the hearing threshold for high frequencies (including 2048 Hz, which was the frequency of the auditory component of our audiovisual targets) and a decrease in capacity for discriminating between frequencies^33,34^. If the audiovisual facilitation loss is due to hearing attenuation, we postulate that vergence, not saccades, would be affected. Audiovisual facilitation occurs only when the two modalities, or the visual target and the locus of the auditory source, are close to each other both in space and time^11,15,16^. Therefore, the mechanism affecting audiovisual facilitation of vergence could be the related to loss of such proximity, namely to poor localization of the sound in depth.

Localization of sound by the brain is the result of complex neural processing. The position of a sound is deduced by analyzing and comparing the two ears' spectral and temporal features. To detect the azimuth of sounds, there must be a comparison of level (ILD, interaural level differences) and time (ITD, interaural time differences) between right and left ear^35^. The mechanisms implied to detect the distance in depth of a sound, situated at the same distance between the right and the left ear, are different and may be more complex than detecting target located laterally. Indeed, the brain is better at determining an azimuth of a sound than its distance in depth^36^. The four cues for sound localization in distance in depth are perceived intensity, direct to reverberant energy ratio (DRR), spectral cues, and dynamics cues^35^. For our study, only the DRR and perceived intensity were able to be perceived by subjects. Dynamic cues were not available as the sound source was stable and, because the buzzer produced a single pure tone of 2048 Hz, there were no spectral cues arising from the buzzer. The perceived intensity is based on the fact that a sound approximately decreases by 6 dB for each doubling of source distance in depth. The direct to reverberant energy ratio (DRR) is the difference between the sound waves directly coming from the sound source (direct energy) and the sound waves reflecting from surfaces before reaching the listener (reverberant energy). Compared to the direct energy, the reverberant sound level varies only slightly with distance in depth^37^. The literature on the effect of hearing loss on auditory distance perception is currently poor; see the article of Kolarik et al.^38^ for review. Akeroyd^39^ demonstrated that hearing loss affects the DRR cues without affecting perceived intensity cues. Therefore, we propose that age-related physiologic hearing loss could explain why our elderly participants could no longer use auditory cues from the targets in depth to increase vergence latency.

To sum up, this study provides new insight on the selective effect aging has on saccades and vergence eye movements; aging affects latency for both movements, amplitude for vergence only, and the average velocity of both saccades and vergence. We argue that the velocity effects are due to age-related slowing of visual processing that guides the execution of the movement rather than motor aging per se. Another major finding is that the existence of multisensory facilitation (for audiovisual vs visual targets) for saccades latency is preserved with age, while such facilitation vanishes for targets in depth; this could be due to physiologic attenuation of hearing capability to localize the sound target in depth along the median plane where no left/right ear differences are present.

## Materials and methods

The study was approved by the ethics committee “Ile de France II” (N° ID RCB: 019-A02602-55, approved the 10/03/2020). All methods were performed in accordance with the relevant guidelines and regulations of Scientific Reports.

### Participant

Participants were divided into two groups: an elderly group (EG), composed of 45 participants aged between 51 and 83 years (mean 65 + 6.9) and a young group (YG) composed of 22 participants aged between 21 and 30 years (mean 25 ± 2.5). The elderly group was recruited by the RISC (Relais d’Information des Sciences Cognitives, France) platform of the CNRS or by contacting associations likely to have people of appropriate ages. Some of them were retired, while others were still working. All persons were functionally independent and came to the laboratory without assistance. We can consider this sample as an average elderly population. The young group was composed of people working in the same building. All these participants had good vision or wore visual correction. No participants showed neurological or psychiatric disorders nor received medication that could affect their sensory and motor functions. No participants had auditory or oculomotor pathology. Informed consent was obtained from all participants after the nature of the procedure had been explained.

### Oculomotor tests

Left saccades, right saccades, convergences, and divergences are tested with protocols run at the REMOBI device (Patent: US885 1669, WO2011073288) first described by Kapoula et al.^40^; eye movements are recorded binocularly with the head-mounted video-oculography device, Pupil Core (Pupil Labs, Berlin), with a frequency measurement of 200 Hz, which is sufficient to get a good recording of saccade velocity^41^.

The REMOBI is a visio-acoustic surface placed at the eye level; 48 LEDs with nominal frequency 626 nm, 180 mCd, and a diameter of 3 mm are embedded at 4 isovergence arcs. The embedded algorithms enable testing of different types of eye movements with the lighting of different sequences of LEDs. Adjacent to each LED is an embedded buzzer that delivers a pure sound at 2048 Hz, with a sound pressure level of 70 dB SPL. The participant sat in front of the REMOBI placed at eye level. His head was not constrained but he is instructed not to move it.

**Figure 4:**
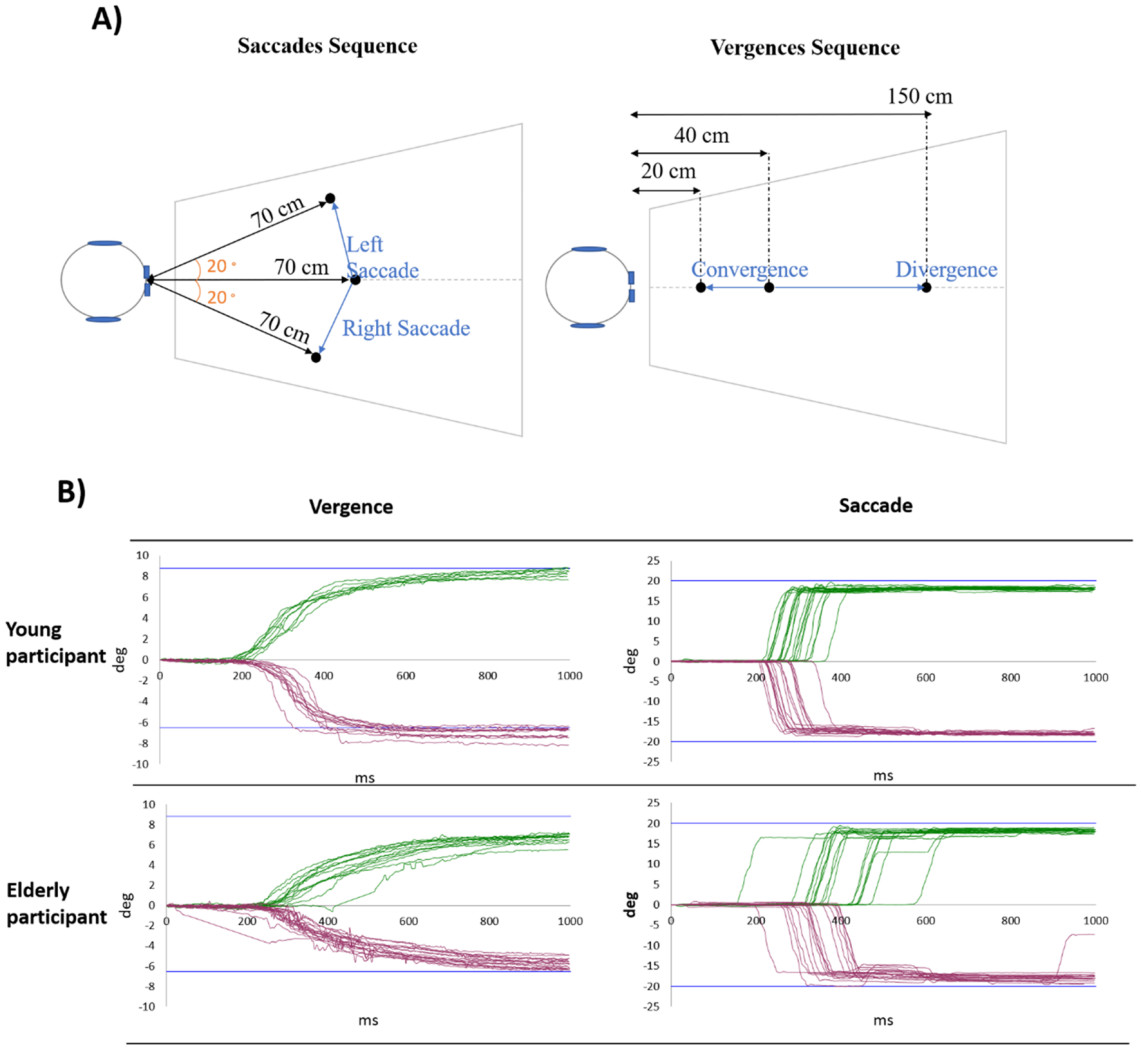
(**A**) Top-view of the position of the LEDs for the saccades sequence (on the left) and for the vergence sequence (on the right). (**B**) Graphic produced by the AIDEAL software, permitting a quick overview of the results for vergence and saccades sequences. The two graphs on the top represent the results for a young adult participant, while the two graphs on the bottom represent the results for an elderly participant. Each curve represents the rotation of the eyes (in degrees on the Y-axis) in function of time (in ms on the X-axis), made during one trial, from the fixation LED to the target LED (0° means that the participant is still looking at the fixation LED). For the vergence sequence, the green upper curves represent the convergence movements and the purples lower curves represent the divergence movements. For the saccade sequence, the green upper curves represent the right saccade movements, and the purple lower curves represent the left saccade movements. The blue lines represent the amplitude required to reach the fixation LED: − 6.50° for the divergence, 8.76° for the convergence, − 20° for the left saccade and 20° for the right saccade.

Two sequences were used in this study: the saccades sequence, measuring left and right saccades, and the vergence sequence, measuring divergence and convergence. The saccades sequence was composed of 20 trials of right saccades and 20 trials of left saccades which were randomly interleaved. The initial fixation LED is located at 70 cm. The angles formed by the two lateral LED targets for saccades were 20° to the left and right. The vergence sequence was composed of 20 trials of divergence and 20 trials of convergence, which were also randomly interleaved. From the participant’s eye, the central initial fixation LED is located at 40 cm with the nearest LED located at 20 cm and the farthest LED located at 150 cm. The convergence movement required an angle of 8.76° and the divergence movement an angle of 6.5°. All these values are given with a pupillary distance of 62 mm. (Fig. 4A) The stimulus was aligned as precisely as possible in the mid-sagittal plane between the eyes to minimize accompanying horizontal saccades for binocular stimulation.

During a trial, the participant first fixated at the central fixation LED for a random time between 1200 and 1800 ms, then a target LED is light on for 2000 ms, following an overlap paradigm (i.e., the central fixation LED switch off 200 ms after the onset of the target LED). Between trials, a blanked period of 300 ms to 700 ms was applied. Participants were instructed to fixate as quickly and as accurately as possible the target LED and to maintain the fixation.

### The two different sensory modalities

The participants were tested with two saccades and two vergence paradigms: one towards audiovisual targets (AV targets) and one toward visual targets (V targets).

During the audiovisual paradigm, the LED lighting was preceded by an auditory warning, i.e., activation of the buzzer adjacent to that LED 50 ms before the LED for a duration of 100 ms. During the visual paradigm, no buzzer sound occurs before the LED.

### The double mode control of vergence

We based our analysis of vergence on the double mode control of vergence. Indeed, its dynamics can be dissected into two components: an initial open-loop component of enhanced speed and a sustaining, closed-loop component, which is slower and driven by visual feedback. This model was first described by Semmlow^25^ and has since been used as a theoretical reference by several studies^42–44^.

In this study, the vergence movement is divided into two parts: the very first part of the vergence eye movement, for a duration more or less equivalent to that of a saccade (about 60 ms), and the visually-driven subsequent portion measuring 160 ms.

The mean duration of saccades is less than 100 ms, which is too short for the visual system to give feedback, rendering saccades as complete open-loop movements^45^. In this way, for both types of movements, we measure the movements done in open-loop mode, i.e., without possible visual feedback. Our 5 deg /sec criteria for onset and offset of vergence enabled us to capture this initial part of the vergence (see below).

### Eye movement analysis

Data recorded with the Pupil Labs eye tracker are analyzed with the AIDEAL software.

For saccades, the whole movement is analyzed. The onset and the offset of the saccades are defined as the time points where the velocity went above or below 10% of the peak velocity; practically, this corresponded to values above or below 40°/s (as the peak velocity of 20° saccades is typically above 400°/s). For these movements, AIDEAL treated the conjugate signal, e.g. the L + R position /2.

For vergence, we analyzed either the open-loop and the close-loop components of vergence with the double mode control model. The beginning and end of the initial open-loop vergence movements are defined as the time point when the eye velocity exceeded or dropped below 5°/s: these criteria are standard and are applied automatically by the AIDEAL software (Patent: PCT/EP2021/062224 7 May 2021). The total vergence movement is measured by adding the subsequent visually-driven period of 160 ms. This period corresponds to the time constant of the extraocular muscles. It should be noted that the slow movement continued after this 160 ms period and this was not included in our analysis. For these movements, the vergence signal was derived by AIDEAL by calculating the difference between the two eyes from the individual calibrated eye position signals (i.e., left eye–right eye). The velocity of the horizontal conjugate and disconjugate signals were computed using a symmetrical two-point differentiator combined to low-pass filtering with a Gaussian FIR filter (cut-off frequency 33 Hz).

Trials from different sessions are regrouped (see below). Trials with blinks were excluded, which occurred frequently for vergence. For divergence, 29% ± 12% trials were excluded for the YG and 26% ± 20% for the EG. For the convergence, 45% ± 14% were excluded for the YG and 38% ± 17% for the EG. For the left saccades, 16% ± 11% were excluded for the YG and 28% ± 17% for the EG. For the right saccades, 9% ± 10% were excluded for the YG and 24% ± 18% for the EG. Outlier values (e.g., values below or beyond 2SD) were also excluded.

Results are given in an excel spreadsheet and with graphs (Fig. 4B).

### Calibration of the pupil labs device

The standard Pupil Labs calibration (Pupil Capture) was applied using a target that was presented at the eyes level with a viewing distance of 1 m. The subjects had to fix on the center of this target and to move slowly their head rightward, downward, leftward and upward. They then repeated the sequence^46^.

### Characteristics measured

The oculomotor characteristics we measured were latency, peak velocity, average velocity and gain amplitude. Latency, expressed in ms, is the time between the onset of the target LED and the onset of the eye movement. Peak velocity, expressed in °/s, was measured for saccades and the initial *open-loop component* of vergence. Average velocity, expressed in °/s, and the gain amplitude, expressed as a percentage of the amplitude required to reach the target (20° for saccades, 8.76° for convergence and 6.5° for divergence), were measured for saccades and the total vergence.

### Statistical analysis

We applied a three-way ANOVA with repeated measures applied separately for the following eye movement characteristics: Latency, peak velocity, average velocity, Gain amplitude.

The dependent variable is the eye movement characteristic; the independent variables are the kind of movement (divergence, convergence, left saccades, right saccades), the target modality used (AV targets or V targets) and the age (Elderly or Young).

## Data Availability

The datasets generated during and/or analyzed during the current study are available from the corresponding author on reasonable request.
