# Publisher Correction: Loss of audiovisual facilitation with age occurs for vergence eye movements but not for saccades

**DOI:** 10.1038/s41598-022-20028-7

**Published:** 2022-09-12

**Authors:** Martin Chavant, Zoï Kapoula

**Affiliations:** IRIS Laboratory, Neurophysiology of Binocular Motor Control and Vision, CNRS UAR 2022, University of Paris, 45 Rue des Saints Pères, 75006 Paris, France

Correction to: *Scientific Reports* 10.1038/s41598-022-08072-9, published online 15 March 2022

The original version of this Article contained errors in the Materials and methods section, where patent numbers were omitted.

As a result, under the sub-heading ‘Oculomotor tests’,

“Left saccades, right saccades, convergences, and divergences are tested with protocols run at the REMOBI device first described by Kapoula et al.^40^; eye movements are recorded binocularly with the head-mounted video-oculography device, Pupil Core (Pupil Labs, Berlin), with a frequency measurement of 200 Hz, which is sufficient to get a good recording of saccade velocity^41^.”

now reads:

“Left saccades, right saccades, convergences, and divergences are tested with protocols run at the REMOBI device (Patent: US885 1669, WO2011073288) first described by Kapoula et al.^40^; eye movements are recorded binocularly with the head-mounted video-oculography device, Pupil Core (Pupil Labs, Berlin), with a frequency measurement of 200 Hz, which is sufficient to get a good recording of saccade velocity^41^.”

Additionally, under the sub-heading ‘Eye movement analysis’,

“The signal is derived by calculating the difference between the two eyes from the calibrated eye position signals (i.e., left eye–right eye). The velocity of the horizontal conjugate and disconjugate signals were computed using a symmetrical two-point differentiator combined to low-pass filtering with a Gaussian FIR filter (cut-off frequency 33 Hz).

For saccades, the whole movement is analyzed. The onset and the offset of the saccades are defined as the time points where the velocity went above or below 10% of the peak velocity; practically, this corresponded to values above or below 40°/s (as the peak velocity of 20° saccades is typically above 400°/s).

For vergence, we analyzed either the open-loop and the close-loop components of vergence with the double mode control model. The beginning and end of the initial open-loop vergence movements are defined as the time point when the eye velocity exceeded or dropped below 5°/s: these criteria are standard and are applied automatically by the AIDEAL software. The total vergence movement is measured by adding the subsequent visually driven period of 160 ms. This period corresponds to the time constant of the extraocular muscles. It should be noted that the slow movement continued after this 160 ms period and this was not included in our analysis.”

now reads:

“For saccades, the whole movement is analyzed. The onset and the offset of the saccades are defined as the time points where the velocity went above or below 10% of the peak velocity; practically, this corresponded to values above or below 40°/s (as the peak velocity of 20° saccades is typically above 400°/s). For these movements, AIDEAL treated the conjugate signal, e.g. the L + R position /2.

For vergence, we analyzed either the open-loop and the close-loop components of vergence with the double mode control model. The beginning and end of the initial open-loop vergence movements are defined as the time point when the eye velocity exceeded or dropped below 5°/s: these criteria are standard and are applied automatically by the AIDEAL software (Patent: PCT/EP2021/062224 7 May 2021). The total vergence movement is measured by adding the subsequent visually driven period of 160 ms. This period corresponds to the time constant of the extraocular muscles. It should be noted that the slow movement continued after this 160 ms period and this was not included in our analysis.

For these movements, the vergence signal was derived by AIDEAL by calculating the difference between the two eyes from the individual calibrated eye position signals (i.e., left eye–right eye). The velocity of the horizontal conjugate and disconjugate signals were computed using a symmetrical two-point differentiator combined to low-pass filtering with a Gaussian FIR filter (cut-off frequency 33 Hz).”

Finally, the Acknowledgements section was incomplete.

“The authors thank Lindsey Ward for providing comments on the manuscript. They thank Julie Bestel, Audilab and ANRT CIFRE for financial support for CHAVANT M.”

now reads:

“The authors thank Lindsey Ward for providing comments on the manuscript. They thank Julie Bestel, Audilab and ANRT CIFRE for financial support for CHAVANT M. Zoi Kapoula obtained patents for the technology used to conduct this experiment: REMOBI table (patent US885 1669, WO2011073288); AIDEAL analysis pending international patent application (PCT/EP2021/062224 7 May 2021).”

The original Article has been corrected.

